# Aptamers Targeting Immune Checkpoints for Tumor Immunotherapy

**DOI:** 10.3390/pharmaceutics17080948

**Published:** 2025-07-22

**Authors:** Amir Mohammed Abker Abdu, Yanfei Liu, Rami Abduljabbar, Yunqi Man, Qiwen Chen, Zhenbao Liu

**Affiliations:** 1 Department of Pharmaceutics, Xiangya School of Pharmaceutical Sciences, Central South University, Changsha 410013, China; 247208001@csu.edu.cn (A.M.A.A.); ramiabduljabbar@csu.edu.cn (R.A.); myqcsu@csu.edu.cn (Y.M.); 2 Department of Pharmaceutical Engineering, College of Chemistry and Chemical Engineering, Central South University, Changsha 410083, China; liuyf@csu.edu.cn (Y.L.); chenqw@csu.edu.cn (Q.C.); 3 Molecular Imaging Research Center, Central South University, Changsha 410008, China

**Keywords:** aptamers, immune checkpoints, cancer immunotherapy

## Abstract

Tumor immunotherapy has revolutionized cancer treatment by harnessing the immune system to recognize and eliminate malignant cells, with immune checkpoint inhibitors targeting programmed death receptor 1 (PD-1), programmed death-ligand 1 (PD-L1), and cytotoxic T-lymphocyte-associated protein-4 (CTLA-4) demonstrating remarkable clinical success. However, challenges such as treatment resistance, immune-related adverse effects, and high costs highlight the need for novel therapeutic approaches. Aptamers, short, single-stranded oligonucleotides with high specificity and affinity for target molecules, have emerged as promising alternatives to conventional antibody-based therapies. This review provides a comprehensive analysis of aptamer-based strategies targeting immune checkpoints, with a particular focus on PD-1/PD-L1 and CTLA-4. We summarize recent advances in aptamer design, including bispecific and multifunctional aptamers, and explore their potential in overcoming immune resistance and improving therapeutic efficacy. Additionally, we discuss strategies to enhance aptamer stability, bioavailability, and tumor penetration through chemical modifications and nanoparticle conjugation. Preclinical and early clinical studies have demonstrated that aptamers can effectively block immune checkpoint pathways, restore T-cell activity, and synergize with other immunotherapeutic agents to achieve superior anti-tumor responses. By systematically reviewing the current research landscape and identifying key challenges, this review aims to provide valuable insights into the future directions of aptamer-based cancer immunotherapy, paving the way for more effective and personalized treatment strategies.

## 1. Introduction

Cancer remains a leading cause of death worldwide. Over the last 5 years, more than 50 million individuals have been diagnosed with cancer. Globally, over 10 million cancer-related fatalities occur annually, and this number is projected to rise to 28–30 million by 2040 [1]. The emergence of cancer immunotherapy in the 1980s made a significant impact. The concept of gaining immunity to specific diseases was explored by Thucydides in the 5th century BC, while the first immune checkpoint molecule CTLA-4 was identified by Brunet and colleagues in 1987, emphasized by the FDA’s approval of the first immunotherapeutic agent, an interferon-α2 inhibitor [2], prompting extensive research into novel treatment strategies. One major challenge in cancer immunotherapy is the emergence of resistance, which can be either intrinsic (present before treatment) or acquired (developing during treatment). Tumors evade immune surveillance by downregulating antigen presentation, upregulating immune checkpoint molecules, or creating an immunosuppressive tumor microenvironment (TME). Some tumors, classified as “hot”, exhibit intense immune infiltration and response, while others, classified as “cold”, show little to no immune activity and are unresponsive to treatment [3]. Given the inherent challenges of tumor immunotherapy, including treatment resistance and immune-related side effects, more precise patient classification techniques are urgently needed. Due to variations in tumor microenvironments and genetic backgrounds, current therapies often yield inconsistent outcomes, making treatment efficacy difficult to predict [4].

These challenges, including immune evasion and therapy resistance, underscore the need for novel strategies to enhance treatment efficacy. One promising approach involves the use of aptamers. Tumor immunotherapy has revolutionized oncology by leveraging the immune system to recognize and eliminate malignant cells. To improve its effectiveness, researchers are exploring various strategies, including the incorporation of aptamers. Aptamers are short DNA or RNA oligonucleotides that bind specifically to target molecules. They hold great promise in cancer treatment, particularly in enhancing immunotherapy, due to their high specificity, reduced side effects, and lower production costs compared to conventional therapies. Recent studies have demonstrated that integrating aptamers into immunotherapeutic approaches can further improve treatment outcomes by precisely targeting key mechanisms involved in cancer progression and immune evasion [5,6].

Tumor immunotherapy represents a paradigm shift in cancer treatment by harnessing the immune system’s ability to combat cancer. Unlike conventional treatments, such as chemotherapy and radiation, which directly target cancer cells but often damage healthy tissues, immunotherapy aims to activate or enhance immune responses specifically against tumors. Its mechanisms include immune checkpoint blockade, T-cell activation, and cytokine therapy. Immune checkpoint inhibitors (ICIs) have been reported to restore anticancer immunity, primarily by regenerating, initiating, and activating cytotoxic T cells, leading to a long-lasting antitumor immune response [7]. For example, immune checkpoint inhibitors target proteins, such as PD-1, PD-L1, and CTLA-4, which tumor cells exploit to evade immune responses [4,8]. By blocking these proteins, checkpoint inhibitors can restore the immune system’s ability to recognize and eliminate cancer cells, significantly improving survival outcomes in various cancers, including melanoma, non-small cell lung cancer, and renal cell carcinoma [9]. The Food and Drug Administration (FDA) approved ipilimumab, a CTLA-4 inhibitor, as the first ICI in 2011 [7]. Despite its success, challenges, such as treatment resistance, immune-related adverse effects, and high costs, highlight the need for innovative approaches, including aptamer-based therapeutics [6].

Studies on immune checkpoint-based aptamers have provided valuable insights into the research landscape. These studies can be categorized based on their immune checkpoint targets, such as PD-1/PD-L1 and CTLA-4, as well as the types of aptamers developed, including bispecific and multifunctional variants. The published studies highlight the prominence of PD-1/PD-L1 research while underscoring the potential for expanding investigations into other immune checkpoints. The research landscape in immune checkpoint-targeted immunotherapy has advanced significantly, with a focus on key immune checkpoints and the development of diverse aptamer types. Among these, PD-1 and PD-L1 have been the most extensively studied, mainly due to their role in immunotherapy. Other checkpoints, such as CTLA-4 and NKG2A, and additional targets like nucleolin and NKG2D, have been explored in various studies. 

Notably, there has been substantial interest in targeting multiple immune checkpoints simultaneously, with the investigation of bispecific checkpoint-targeting strategies. In terms of aptamer utilization, PD-1/PD-L1 aptamers have been the most frequently employed. Bispecific aptamers, which target multiple checkpoints, are also gaining significant attention, with studies examining their application. Other aptamer types, such as those targeting CTLA-4 and nucleolin, are less commonly studied. The growing interest in multifunctional aptamers suggests a trend toward developing more sophisticated and targeted therapeutic strategies in cancer immunotherapy.

Aptamers are synthetic oligonucleotides with unique properties that enable them to bind specific target molecules with high specificity and affinity. Often referred to as “chemical antibodies”, aptamers offer significant advantages over conventional antibodies in therapeutic applications. They are smaller in size, easier to chemically modify, and less immunogenic than antibodies, making them highly versatile in clinical settings. Their small size enhances tissue penetration, while site-specific chemical modifications, such as PEGylation, improve therapeutic efficacy and stability. For example, Pegaptanib (Macugen), an FDA-approved aptamer for the treatment of age-related macular degeneration, highlights the clinical potential of aptamers and their reduced immunogenicity. In cancer immunotherapy, aptamers like AS1411, which target nucleolin, have shown promise in inhibiting tumor growth and metastasis in clinical trials for breast and lung cancer. Similarly, NOX-A12 (Olaptesed Pegol), which targets CXCL12, has demonstrated potential in disrupting tumor microenvironment interactions and enhancing the efficacy of immune checkpoint inhibitors in early-phase trials. These advancements underscore the significant role of aptamers in transforming cancer therapeutics [10].

Despite significant progress in aptamer-based tumor immunotherapy, challenges remain in optimizing their clinical application, enhancing stability, and improving targeted delivery. This review aims to provide a comprehensive analysis of the current advancements in aptamer-based immunotherapy, with a particular focus on their role in targeting immune checkpoints, such as PD-1, PD-L1, and CTLA-4. We will explore the latest developments in aptamer design, including bispecific and multifunctional aptamers, and discuss their potential in overcoming immune resistance and enhancing treatment efficacy. Additionally, we will highlight strategies for improving aptamer stability, bioavailability, and tumor penetration through chemical modifications and conjugation with nanoparticles or immune-stimulatory agents. By systematically reviewing existing studies and identifying key challenges, this review seeks to provide valuable insights into the future directions of aptamer-based cancer immunotherapy, paving the way for more effective and personalized treatment strategies.

### 1.1. Aptamer Binding Targets in Cancer Immunotherapy

Furthermore, aptamers exhibit high stability under various conditions and can be cost-effectively produced, making them particularly attractive for use in cancer immunotherapy. Cancer cells often evade immune surveillance by exploiting immune checkpoint receptors, such as TIM-3, LAG-3, CTLA-4, and PD-1. Aptamers can specifically bind to these receptors, blocking inhibitory signals that suppress T-cell activity and restoring T-cell function to recognize and eliminate malignant cells [4]. In tumor immunotherapy, aptamers have demonstrated potential in targeting key immune checkpoints, such as PD-1 and PD-L1, which are critical regulators of immune responses in cancer. Studies have shown that aptamers can block these checkpoints. This restores T-cell activity and enhances anti-tumor immunity [5]. In a separate study, an anti-CTLA-4 aptamer was shown to block CTLA-4, preventing the suppression of co-stimulatory signals necessary for T-cell proliferation and increasing T-cell activation [11]. Li and colleagues found that aptamers help maintain T-cell stemness and reduce depletion by blocking inhibitory checkpoints, as evidenced by the downregulation of exhaustion-associated gene sets in T cells. Additionally, for long-lasting anti-tumor responses, aptamers can mimic co-stimulatory signals, such as CD28, to promote T-cell activation and memory formation [12]. Moreover, aptamer-based therapies can be conjugated with various therapeutic agents, including nanoparticles or chemotherapeutic drugs, to deliver targeted treatments with reduced systemic toxicity. Camorani et al. highlighted the potential of aptamer-based therapies to enhance immune checkpoint blockade in triple-negative breast cancer, demonstrating a novel approach to overcoming treatment resistance and achieving better clinical outcomes for cancer patients [13]. Compared with antibodies, aptamers offer a distinct advantage due to their ability to penetrate tumors and bind to intracellular targets with high affinity and specificity. Additionally, their synthetic nature allows for rapid production and customization to target various types of cancer. These properties make aptamers valuable tools in advancing tumor immunotherapy, providing opportunities for personalized and cost-effective cancer treatments. As research continues to explore their full potential, aptamers are expected to play a crucial role in the future of cancer therapeutics [6,12].

Aptamers often exhibit high binding affinities to their targets, typically in the nanomolar to picomolar range. Their distinct three-dimensional architectures enable strong interactions with target molecules through hydrogen bonding, van der Waals forces, and electrostatic interactions [14,15]. Various aptamers have shown strong binding affinities to key immune checkpoints, including PD-1, PD-L1, NKG2A, and CTLA-4. Binding affinities are expressed in nanomolar (nM) concentrations, where a lower value indicates stronger affinity. These binding affinities are crucial for evaluating the effectiveness of aptamers in modulating immune checkpoint activity, a key factor in cancer immunotherapy. Heatmaps generated from these data can help identify aptamers with the strongest potential to block immune checkpoints and thereby enhance immune system activation within TME. The binding affinity of an aptamer depends on its secondary and tertiary structure, nucleotide sequence, and target molecule. For example, aptamers with specific stem–loop configurations often exhibit enhanced binding affinity [16]. However, factors within the TME, such as a dense extracellular matrix, hypoxia, and limited accessibility of target molecules (e.g., cell surface receptors or proteins), can influence aptamer binding efficacy. By selectively binding to overexpressed receptors or proteins on cancer cells (e.g., EGFR, nucleolin, or PD-L1), high-affinity aptamers can facilitate targeted drug delivery or imaging [17]. Sun et al. reported that the TME is characterized by low pH, hypoxia, and high concentrations of nucleases, which can degrade aptamers or reduce their binding affinity. Chemical modifications (e.g., incorporation of 2′-O-methyl or 2′-fluoro groups) have been shown to enhance aptamer stability and binding affinity under physiological conditions [18]. Liu et al. spotlighted the relationship between aptamer binding affinities and variations in aptamer structures. These structures have stems and various loops such as hairpin loops, inner loops, quadruplex loops, inner loop pseudoknots, and G-quadruplex structures. Adjusting the aptamer’s structure has a direct effect on binding stability [15].

Binding affinities of aptamers are typically measured using techniques, such as electrophoretic mobility shift assay (EMSA), isothermal titration calorimetry (ITC), and surface plasmon resonance (SPR). SPR, for example, has been used to assess the binding of an aptamer targeting vascular endothelial growth factor (VEGF), yielding a dissociation constant (Kd) of ~50 pM [19]. These methods provide quantitative insights into the strength of aptamer–target interactions. In therapeutic applications, binding affinity is critical for aptamer efficacy. For example, the thrombin-binding aptamer, which has a nanomolar-range dissociation constant, has demonstrated strong binding to thrombin [20]. Higher binding affinity directly enhances therapeutic efficacy by ensuring precise targeting of immune checkpoints, such as PD-1 and CTLA-4, within the TME. For instance, the higher affinity of an aptamer for multiple checkpoints could improve its ability to regulate immune responses, making it more effective in overcoming tumor-induced immune suppression.

Aptamer interactions with various immune cells, including B cells, natural killer (NK) cells, T cells, and macrophages, can be categorized as high, moderate, or low, based on their degree of immune activation or modulation. For example, a specific aptamer may exhibit strong interactions with T cells and moderate interactions with macrophages, suggesting its potential to enhance T-cell-mediated anti-tumor immunity while also influencing macrophage behavior. In contrast, another aptamer may show low interaction with T cells but high interaction with B cells and macrophages, indicating its role in modulating humoral immune responses or tumor-associated inflammation. These interaction patterns are essential for understanding how aptamers influence immune responses in cancer therapy, particularly in enhancing immune cell activation or inhibiting tumor growth. Heatmaps based on these data will provide a visual representation of how aptamers affect different immune cell types, further guiding their potential therapeutic applications. Several aptamers have been designed for cancer immunotherapy to target specific molecules implicated in immune evasion (Table 1). These aptamers have a variety of functions, including inhibiting immunological checkpoints and activating T cells.

### 1.2. The Mechanisms of Aptamer Enhancing Immune Responses

Cancer immunotherapy was developed based on the observation that tumor cells differ from healthy cells in both the quantity and composition of their proteins and antigen repertoires. Immune cells recognize tumor-associated antigens (TAAs) as foreign, triggering an immune response against tumor cells. Ideally, this response would be strong enough to eliminate the tumor. However, in most cases, the immune reaction is insufficient, and worse, tumors gradually develop defense mechanisms that allow them to evade immune surveillance. These mechanisms include the production of inhibitory ligands that suppress immune function through negative co-stimulation [34,35]. Enhancing tumor immune responses is a key therapeutic goal in cancer immunotherapy. Strategies to improve therapeutic efficacy include limiting undesirable immune suppression and providing stimulatory signals to boost anti-tumor immunity. Traditionally, therapeutic approaches have focused on targeting immune receptors, cytokines, and chemokines using antibodies, soluble receptor ligands, or recombinant cytokines.

Aptamers show the ability to interact with the immune system. Most of the studies were designed to suppress immune responses and were intended for the treatment of autoimmune diseases rather than cancer. The application of aptamers in cancer immunotherapy, particularly for enhancing anti-tumor immunity, remains an area of emerging interest. Here, we highlight key studies that demonstrate how aptamers modulate immune responses to enhance immunotherapy. For instance, aptamers can stimulate innate immune responses to improve vaccine efficacy by facilitating antigen presentation and T cell activation. They achieve this by binding to pattern recognition receptors (PRRs) on dendritic cells or macrophages. For example, CpG-containing DNA aptamers can activate Toll-like receptor 9 (TLR9), leading to the production of pro-inflammatory cytokines and an enhanced immune response [36,37]. Additionally, studies have shown that aptamers can be engineered to selectively bind immune molecules or cells, including cytokines, B cells, and T cells, to modulate immune responses. Aptamers targeting immune checkpoint molecules, such as PD-1 or CTLA-4, can enhance anti-tumor immunity by blocking inhibitory signals that suppress T cell activation [17,38]. Similarly, aptamers can prevent infection or immune evasion by disrupting interactions between pathogens (bacteria or viruses) and the immune system. For example, Dey and colleagues developed an aptamer targeting the HIV-1 gp120 protein to inhibit viral attachment and entry [39]. Furthermore, aptamers have demonstrated potential in modulating inflammatory responses by binding to cytokines, chemokines, or their receptors. For instance, aptamers targeting TNF-α or IL-6 can inhibit pro-inflammatory signaling pathways. A notable example is Pegaptanib, an RNA aptamer used to treat age-related macular degeneration by targeting VEGF, thereby reducing inflammation and angiogenesis [40]. In autoimmune diseases, aptamers can be employed to suppress aberrant immune responses by targeting autoantibodies or immune cells involved in disease pathogenesis. Wu and colleagues investigated the use of aptamers against IFN-γ for the treatment of rheumatoid arthritis, demonstrating their potential in autoimmune therapy [41]. These findings highlight the versatility of aptamers in modulating immune responses and make them promising tools for immunotherapy and immune-related disease treatment.

**Figure 1:**
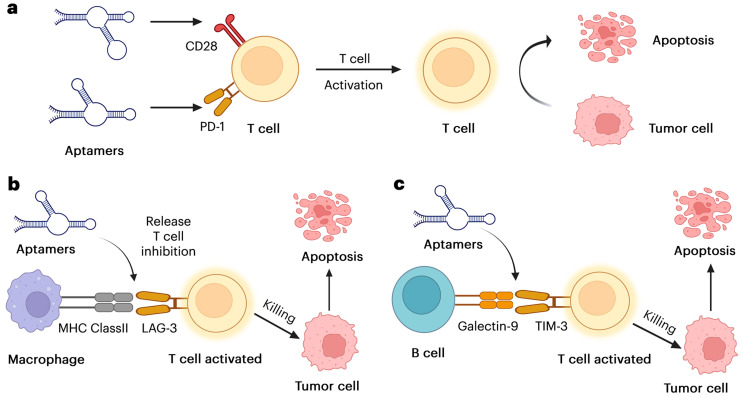
(**a**) Aptamers are designed to bind to specific receptors on immune cells, such as T-cell surface receptors (e.g., CD28 or PD-1). By binding to these receptors, aptamers can either activate or inhibit signaling pathways that regulate immune cell function. (**b**) Aptamer targeting LAG-3 on T-cell surface to prevent the interaction with MHC class II molecules on macrophages to release T-cell inhibition, to attack tumor cells. (**c**) TIM-3 aptamers are created to inhibit the interaction between TIM-3 on a T-cell surface and galectin-9 on a B cell, reinvigorating fatigued T cells and increasing anti-tumor responses.

Cancer cells with genetic and epigenetic abnormalities produce a wide range of tumor-associated antigens, which the immune system recognizes as non-self and targets them for elimination. However, cancer cells have developed multiple defense mechanisms to evade immune destruction and modify the tumor microenvironment to their advantage, promoting tumor growth, invasion, and metastasis. Cancer immunotherapy aims to enhance or restore the immune system’s ability to recognize and eliminate cancer cells by overcoming these immune evasion strategies. In recent years, significant progress has been made in developing aptamer-based approaches to shift the immune system toward an antitumor state, particularly in triple-negative breast cancer (TNBC). Emerging evidence suggests that aptamers can recruit immune cells to tumor sites, block immune checkpoint pathways, and enhance the cytotoxic activity of immune cells, making them promising candidates for cancer immunotherapy [42]. Figure 1 illustrates how aptamers, which are synthetic nucleic acid molecules, bind to certain targets, such as tumor antigens or immune checkpoints, to enhance immune responses by blocking inhibitory signals and restoring T-cell activity, thereby improving anti-tumor immunity.

This emphasizes how aptamers can be used as immunomodulatory agents in cancer treatment by reviving repressed T-cell activity. To enhance clinical applications, further studies should focus on aptamer optimization, combination therapy, and in vivo effectiveness.

#### 1.2.1. Aptamer Direct NK Cells and Macrophages to the Tumor

**Figure 2:**
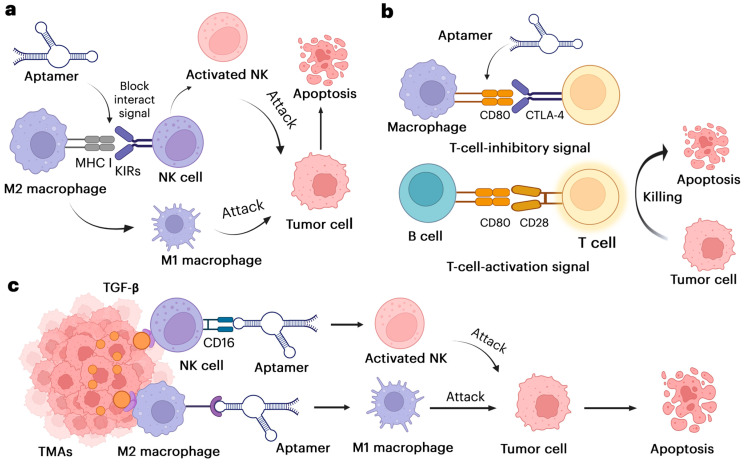
The figure illustrates the function of NK cells and macrophages in the tumor microenvironment (TME) and how aptamers restore their activity. (**a**) Aptamer targeting KIRs receptor on NK cells to block the interaction with MHC class I molecules on macrophages, then activate NK cells and promote macrophages against tumor cells. (**b**) Aptamer designed to target CTLA-4 prevents the binding of B7-1 (CD80) on antigen-presenting cells (APCs), such as macrophages and B cells, to the CTLA-4 receptor on T-cell surfaces. This aptamer-CTLA-4 binding can restore T-cell activity. (**c**) Aptamers direct both NK cells and macrophages to the tumor site by recognizing and binding to the CD16 receptor on NK cells and the CD206 receptor on macrophages, ultimately contributing to tumor cell destruction.

In the TME, NK cells and macrophages are essential components that, depending on their activation status and interactions with other cells, can either promote or inhibit tumor growth. Aptamers targeting these immune cells and altering their activity have shown promising results. NK cells, as natural immune effectors, identify and destroy tumor cells by releasing cytotoxic molecules (e.g., TNF-α, and FasL) and generating cytokines such as IFN-γ. However, tumor-derived substances, such as prostaglandins, adenosine, and TGF-β, can inhibit NK cell activation in the TME, leading to immune evasion. Macrophages in the TME primarily exist in two phenotypes: M2 (pro-tumor) and M1 (anti-tumor). While M2 macrophages promote tumor growth, angiogenesis, and immune suppression, M1 macrophages foster inflammation and induce tumor cell death by releasing cytotoxic molecules (e.g., IL-1β, IL-6, caspases). Typically, tumor-associated macrophages (TAMs) are M2-like and support tumor progression [43]. In the TME, aptamers can be designed to target immune or tumor cells specifically. By binding to receptors on NK cells or macrophages (e.g., CD206 on M2 macrophages or CD16 on NK cells), aptamers can reprogram their function or direct their migration toward the tumor [44]. Figure 2 below explains how aptamers bind to specific receptors on immune cells (such as NK cells and macrophages), guiding them to the tumor site in the tumor microenvironment and promoting immune-mediated tumor destruction.

Aptamers targeting immune cells such as NK cells and macrophages can address and overcome immunosuppressive challenges by accurately directing immune cells to tumors and enhancing immunotherapy. Additional investigations will improve aptamer design, assess effectiveness in advanced tumor models, and investigate combination therapy to boost anti-tumor immune responses.

#### 1.2.2. Aptamer Alters Immune Cells and Delivers Immune-Modulating Agents

Aptamers have been investigated for their ability to directly alter immune cell function, target tumor-specific antigens, and deliver immune-modulating agents. Tumor-specific antigens (e.g., EGFR, HER2) are proteins or other molecules primarily found on cancerous cells, and aptamers can be engineered to recognize and bind to these molecules. Once bound, aptamers can deliver immune-modulating substances (e.g., IL-2, anti-CTLA-4, anti-PD-L1, anti-PD-1, IFN-α) directly to the tumor site, including cytokines, chemotherapeutic agents, and immune checkpoint inhibitors. This targeted approach enhances therapeutic efficacy and reduces off-target effects [45]. Additionally, aptamers can directly alter immune cell activity by binding to receptors or molecules on immune cells, such as T cells, to improve anti-tumor immune responses. For example, aptamers can be designed to activate stimulatory pathways or block inhibitory immune checkpoints like PD-1 or CTLA-4 [38]. A two-uracil (UU) nucleobase linker is used to fuse albumin-binding aptamers to a Dicer-substrate double-stranded RNA molecule to construct an aptamer-siRNA chimera. The siRNA sense strand is annealed, and albumin-binding aptamer–siRNA chimeras improve siRNA bioavailability to enhance antitumor immunity [46].

Aptamers have tremendous potential for altering immune cell behavior and delivering immuno-modulating substances with high specificity, providing innovative treatment methods for diseases such as cancer, autoimmune disorders, and infections. Their flexibility, low immunogenicity, and simplicity of customization make them viable alternatives to antibodies. However, further research should focus on certain points, such as optimizing stability and delivery, combination therapies, and clinical translation. By addressing these issues, aptamer-based immunotherapy has the potential to be a game changer in medicine.

## 2. PD-1/PD-L1 Pathway and Aptamer Development

**Figure 3:**
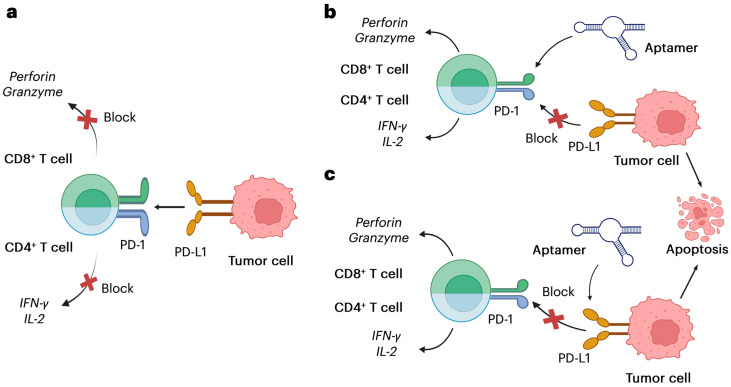
The PD-1/PD-L1 route of action and the use of an aptamer to target PD-1/PD-L1 to block their interaction. (**a**) PD-1 binds to PD-L1 and an inhibitory signal is transmitted to the T-cell, suppressing the immune response and allowing the tumor cell to evade immune detection. (**b**,**c**) Aptamers bind to either PD-1 on T-cell surface or PD-L1 on tumor surface, blocking the PD-1/PD-L1 interaction, restoring T-cell immune activity, and releasing cytotoxic agents (e.g., Perforin, Granzyme, IFNy, and IL-2) to promote cancer cell destruction.

The PD-1/PD-L1 axis plays a crucial role in immune regulation and tumor immune evasion, making it a key target in the field of immunotherapy. The development of aptamers targeting this pathway offers a unique approach to cancer treatment, leveraging their high specificity and versatility. The biological functions of the PD-1/PD-L1 axis and recent advancements in aptamer development for targeting this immune checkpoint are discussed in detail [6,38]. Figure 3, below, clearly illustrates how aptamers can be used to inhibit the PD-1/PD-L1 pathway, enhancing cancer cell killing and restoring immune function.

Targeting the PD-1/PD-L1 pathway, which regulates immune checkpoints, has revolutionized cancer immunotherapy. High-affinity nucleic acid ligands like aptamers can inhibit PD-1/PD-L1 interactions without antibodies and offer improved tissue penetration, lower immunogenicity, and easy customization. Aptamers can boost antitumor immune responses. However, optimizing aptamer design and overcoming tumor resistance mechanisms is still a significant challenge.

### 2.1. The PD-1/PD-L1 Immune Checkpoint Axis

The PD-1 receptor and its ligand PD-L1 play a crucial role in maintaining immune homeostasis by regulating T-cell activity. PD-1, expressed on activated T cells, interacts with PD-L1, which is found on various cells, including tumor cells and immune cells [47,48]. This interaction suppresses T-cell proliferation, cytokine production, and cytotoxic activity, preventing autoimmunity and excessive inflammation under normal physiological conditions [8,49]. However, tumor cells exploit this mechanism by overexpressing PD-L1, thereby creating an immunosuppressive microenvironment that enables them to evade immune surveillance.

This immune checkpoint axis has become a key target in cancer therapy. Immune checkpoint inhibitors, such as monoclonal antibodies targeting PD-1 or PD-L1, have demonstrated remarkable clinical success in restoring T-cell-mediated defenses against tumors [9]. PD-1 was identified by Tasuku Honjo and collaborators in 1992 as a gene associated with apoptosis. Nevertheless, it was confirmed that PD-1 overexpression was not vital for apoptosis; PD-L1 expression on tumor cells, as evaluated by immunohistochemistry (IHC), has emerged as the most widely studied biomarker for leading PD-L1/PD-1 inhibitor therapy. Even so, not all patients derive benefit from PD-L1/PD-1 blockade, necessitating the identification of predictive biomarkers for patient selection. Additionally, resistance mechanisms to PD-L1 and PD-1 inhibition are significant challenges in cancer immunotherapy [2]. In TNBC, (PD-1)/PD-L1 ICBs have shown great promise. In 2020, the FDA approved the use of anti-PD-1 pembrolizumab combined with nab-paclitaxel as a first-line treatment for metastatic TNBC, but the clinical advantage of ICBs in TNBC is inconsistent, with response rates ranging from 15 to 60%, regardless of whether used as monotherapy or in combination. This variability is in part due to the complex and dynamic nature of the TME, which effects breast cancer progression and treatment outcomes. Reliable biomarkers to predict therapeutic efficacy remain limited, making it challenging to optimize their use in breast cancer [50].

However, these treatments also present challenges, including high costs, therapy resistance, and immune-related adverse events, necessitating the exploration of alternative strategies such as aptamer-based therapeutics [6].

### 2.2. Aptamers Targeting PD-1/PD-L1

Aptamers offer an innovative approach to overcoming the limitations of conventional therapies targeting the PD-1/PD-L1 pathway. These short oligonucleotides are designed to bind to their targets with high specificity and affinity, making them ideal candidates for disrupting the PD-1/PD-L1 interaction. Due to their smaller size compared to antibodies, aptamers exhibit superior tissue penetration, including within dense tumor microenvironments. Additionally, their synthetic nature allows for precise chemical modifications, enhancing stability and bioavailability while minimizing immunogenicity [10,51].

Recent advancements in aptamer design have focused on optimizing their binding affinity and specificity to the PD-1/PD-L1 axis. For example, structure-based approaches have been employed to improve aptamer binding efficiency. One notable strategy involves developing aptamers that mimic the PD-1 receptor to competitively inhibit PD-L1 binding to PD-1. These aptamers effectively restore T-cell activity and promote anti-tumor immunity in preclinical models [5]. Preclinical studies have demonstrated the potential of PD-1/PD-L1-targeting aptamers in combination therapies. For instance, integrating aptamers with nanoparticle-based delivery systems has been shown to enhance therapeutic efficacy while minimizing off-target effects. Camorani et al. reported significant tumor regression in mouse models of triple-negative breast cancer using PD-L1-targeting aptamers conjugated with immune-stimulatory agents [52].

Furthermore, early-phase clinical trials are investigating the safety and efficacy of aptamers targeting PD-1/PD-L1. Although these studies remain in their early stages compared to antibody-based therapies, preliminary findings suggest promising outcomes with reduced adverse effects. These results highlight the potential of aptamers to reshape the field of immunotherapy by addressing the limitations of existing treatments and paving the way for personalized therapeutic strategies [53].

### 2.3. Comparison of PD-1/PD-L1 Aptamers with Monoclonal Antibodies

The therapeutic landscape targeting the PD-1/PD-L1 immune checkpoint axis has been dominated by monoclonal antibodies (mAbs), which have revolutionized cancer treatment. However, aptamers have emerged as a promising alternative, offering significant advantages in terms of cost, safety, and therapeutic efficacy. Below, these aspects are examined in detail, followed by a comprehensive comparison table.

#### 2.3.1. Therapeutic Efficacy

Both mAbs and aptamers effectively block the interaction between PD-1 and PD-L1, restoring T-cell activity and enhancing anti-tumor responses. Monoclonal antibodies such as pembrolizumab and nivolumab have demonstrated remarkable success in clinical applications, yielding durable responses in various cancers, including melanoma and non-small-cell lung cancer [6,54]. However, aptamers showcase numerous specific benefits. Due to their diminutive size, aptamers can infiltrate tissues more successfully, undoubtedly growing therapeutic efficacy in dense tumor microenvironments where antibodies often encounter limited access to cancer cells. Moreover, aptamers may be chemically modified to improve their stability and binding affinity. This flexibility permits the improvement of especially tailored molecules that keep activity in challenging physiological situations. Preclinical studies have demonstrated that aptamers targeting PD-L1 can achieve similar or even superior tumor growth inhibition in animal models in comparison to monoclonal antibodies (mAbs), with the added benefit of decreased immunogenicity [5]. Notably, the FDA-approved drug Pegaptanib (Macugen) serves as a prime example of how aptamers have demonstrated therapeutic potential, particularly in the treatment of age-related macular degeneration [40,55].

#### 2.3.2. Safety Profiles

Safety is a crucial concern in immunotherapy, as immune checkpoint blockade can lead to immune-related adverse events (irAEs). Despite their efficacy, monoclonal antibodies are associated with severe irAEs, including pneumonitis, colitis, and endocrinopathies, resulting from excessive immune activation [8]. Cytokine Release Syndrome (CRS), a systemic inflammatory response triggered by cytokine release following immune activation, and hypersensitivity reactions such as fever, chills, and hypotension, are significant challenges associated with mAbs administration [56,57]. Moreover, the toxicity linked to immune checkpoint inhibitors varies by agent. For instance, organ-specific inflammation has been reported with anti-CTLA-4 (ipilimumab) and anti-PD-1/PD-L1 antibodies (pembrolizumab, nivolumab). Additionally, patients receiving mAbs may develop anti-drug antibodies (ADAs), which can reduce drug efficacy and potentially cause allergic reactions [58]. In contrast, aptamers provide a more targeted approach with minimal off-target effects. Their non-proteinaceous nature significantly lowers the risk of immunogenicity and hypersensitivity reactions, making them particularly advantageous for patients with autoimmune disorders or sensitivities to biologics. Furthermore, aptamers are rapidly cleared from circulation when not bound to their target, minimizing prolonged systemic effects [10,59].

#### 2.3.3. Cost Implications

The high cost of monoclonal antibody production remains a significant barrier to widespread adoption, particularly in low- and middle-income countries. mAbs require complex manufacturing processes involving cell cultures and stringent quality control measures, driving up costs. In contrast, aptamers are synthesized chemically, offering a more scalable and cost-effective production process [60]. Additionally, aptamers have shorter development timelines due to their entirely synthetic nature, further contributing to their economic viability. These cost advantages make aptamer-based therapies more accessible, addressing equity issues in cancer care while reducing the financial burden on healthcare systems.

#### 2.3.4. Clinical Applications

Monoclonal antibodies have been widely used in the treatment of infectious diseases, autoimmune disorders, and oncological conditions. Examples include trastuzumab (Herceptin) for breast cancer and adalimumab (Humira) for rheumatoid arthritis. Aptamers, on the other hand, are emerging as promising diagnostic and therapeutic agents. For instance, the FDA-approved aptamer Pegaptanib (Macugen) is used to treat age-related macular degeneration (AMD). Moreover, aptamers are actively being investigated for cancer treatment and precision drug delivery [40,61].

These advantages highlight why aptamers are increasingly being explored as complementary or alternative agents to antibodies in research, therapy, and diagnostics. As illustrated in Table 2, the comparative analysis points out the key differences between PD-1/PD-L1 aptamers and monoclonal antibodies. Aptamers have various advantages over monoclonal antibodies, including smaller size and lower immunogenicity.

In cancer immunotherapy, PD-1/PD-L1 aptamers have great potential as substitutes for monoclonal antibodies (mAbs). Smaller size, less immunogenicity, simpler modification, and perhaps cheaper production costs are some benefits of aptamers. Aptamers have similar binding affinities and inhibitory effects in preclinical tests, despite mAbs’ well-established effectiveness and safety profiles. To enhance their stability, pharmacokinetics, and in vivo performance, more optimization is necessary.

## 3. Other Immune Checkpoints and Associated Aptamers

Immune checkpoints are essential molecules in the immune system that regulate self-tolerance and prevent overreactions that could lead to tissue damage or autoimmunity. Typically produced on immune cells like T cells, these checkpoints interact with specific ligands on tumor cells or antigen-presenting cells (APCs). In normal circumstances, immune checkpoints regulate the immune response, ensuring it is targeted and balanced. However, tumor cells often exploit these pathways to avoid immune recognition and elimination.

Immune checkpoint therapy that targets cytotoxic T-lymphocyte-associated protein 4 (CTLA-4) and programmed cell death protein 1 (PD-1) significantly increased the survival of melanoma patients. Nevertheless, only a small percentage of patients benefit from these treatments; roughly two-thirds of patients have primary or secondary resistance. Immunological checkpoint blockade resistance research focuses on both extrinsic and intrinsic mechanisms, including alternative immunological checkpoints such as T-cell immunoglobulin and mucin domain-containing protein 3 (TIM-3) and lymphocyte activation gene 3 (LAG-3) [62].

Immunotherapy has revolutionized cancer treatment, with immune checkpoints playing a crucial role in regulating immune responses and enabling ionized cancer treatment and tumor immune evasion. Beyond the PD-1/PD-L1 axis, other immune checkpoints, such as TIM-3, CTLA-4, and LAG-3, have gained significant attention for their roles in tumor immunology. Recent advancements in aptamer technology offer a promising approach to modulating these checkpoints effectively.

### 3.1. Immune Checkpoints and Tumor Immune Evasion

Immune checkpoints like PD-1/PD-L1, TIM-3, LAG-3, and CTLA-4 are critical in tumor immune evasion, as they inhibit T-cell activation, enabling tumors to bypass immune surveillance. For example, PD-1 on T cells interacts with PD-L1 on tumor cells, suppressing immune responses. As highlighted by Pardoll et al., the inhibition of T-cell activation, proliferation, and cytokine production caused by the binding of PD-1 to PD-L1 results in T-cell exhaustion and immune evasion [63]. Similarly, CTLA-4 competes with CD28 for interaction with B7 molecules, reducing T-cell priming. Leach et al. reported that CTLA-4 produces an immunosuppressive tumor microenvironment by delivering inhibitory signals that reduce T-cell activity and enhance regulatory T-cell (Treg) function [64].

The immune evasion mechanisms of TIM-3 and LAG-3 differ slightly. Exhausted T lymphocytes and other immune cells express TIM-3, which binds to ligands such as HMGB1, CEACAM-1, and galectin-9, leading to T-cell dysfunction and death. For example, Anderson et al. found that tumors use TIM-3 to inhibit T-cell-mediated immunity and increase tolerance [65]. LAG-3, expressed on T lymphocytes, has a stronger affinity for MHC class II molecules than CD4. Andrews et al. reported that LAG-3 suppresses T-cell activation and cytokine production, contributing to T-cell exhaustion. Tumors exploit this pathway to avoid immune detection, as LAG-3 and PD-1 are frequently co-expressed [66]. Aptamers targeting these immune checkpoints can reactivate anti-tumor immunity, making them highly relevant for immunotherapy.

### 3.2. CTLA-4 Aptamers

Cytotoxic T-lymphocyte-related antigen 4 (CTLA-4) is one of the earliest immune checkpoints identified and plays a pivotal role in maintaining immune homeostasis. CTLA-4 competes with CD28 for binding to B7 ligands on antigen-presenting cells, suppressing T-cell activation and proliferation. While this mechanism is crucial for preventing autoimmunity, tumors exploit it to evade immune destruction. Inhibitors targeting CTLA-4, such as ipilimumab, have shown success in the cancer immunotherapy field, particularly for skin cancer (melanoma).

Aptamers targeting CTLA-4 offer a unique therapeutic approach by specifically disrupting its interaction with B7 ligands, thereby restoring T-cell activity. Research is increasingly focused on enhancing the stability and specificity of CTLA-4 aptamers. For example, Bertrand et al. highlighted efforts to develop RNA-based aptamers that show improved tumor penetration compared to monoclonal antibodies. Additionally, CTLA-4 aptamers demonstrate lower immunogenicity, addressing a common concern associated with antibody-based therapies [6]. This feature is particularly beneficial for patients requiring repeated or prolonged treatments.

### 3.3. TIM-3 and LAG-3 Aptamers

T-cell immunoglobulin and mucin-domain containing-3 (TIM-3) and lymphocyte-activation gene 3 (LAG-3) are emerging checkpoints involved in tumor-induced immune suppression [38]. TIM-3 interacts with its ligand, galectin-9, to inhibit T-cell receptor signaling and trigger T-cell exhaustion, a common feature in chronic infections and cancer. Similarly, LAG-3 binds to major histocompatibility complex class II (MHC II) molecules, suppressing antigen-specific T-cell responses. Tumor-infiltrating lymphocytes frequently co-express TIM-3 and LAG-3 with PD-1. Gene expression of TIM-3 and LAG-3 was increased in the T-cells of mice that acquired resistance to anti-PD-1 therapy in a lung adenocarcinoma model [65]. The development of aptamers targeting TIM-3 and LAG-3 has opened new avenues in cancer immunotherapy. TIM-3 aptamers are designed to inhibit the TIM-3/galectin-9 interaction, reinvigorating exhausted T cells and enhancing anti-tumor responses. Garcia Melian et al. reported promising preclinical results demonstrating the effectiveness of TIM-3 aptamers in combination treatments with PD-1 inhibitors [10]. LAG-3 aptamers are also gaining attention due to their potential to modulate immune suppression within the tumor microenvironment. Unlike monoclonal antibodies (MAbs), aptamers targeting LAG-3 can be chemically engineered for more potent tumor penetration and prolonged action. Ayass et al. emphasized the potential of these aptamers to synergize with other checkpoint inhibitors to improve therapeutic outcomes [5].

In cancer immunotherapy, aptamers targeting immunological checkpoint molecules, including CTLA-4, TIM-3, and LAG-3, might replace monoclonal antibodies. A preclinical study shows that aptamers can prevent T-cell-inhibitory signals, restoring anti-tumor immunity. Improved pharmacokinetics, binding affinity, and in vivo efficacy are still challenges. Next, research must translate preclinical findings into clinical applications that may lead to innovative cancer immunotherapy tools.

### 3.4. Immune Checkpoints Aptamer Clinical Progress

Targeting immune checkpoint pathways, immune checkpoint aptamers represent a promising class of therapeutic agents that resemble monoclonal antibodies but offer distinct advantages, such as simpler synthesis, lower immunogenicity, and smaller size. Here, we provide an overview of recent developments in immune checkpoint aptamers in preclinical and clinical trials. Among the most researched immune checkpoint treatments are PD-1/PD-L1 inhibitors, and aptamers targeting these pathways have shown potential for enhancing T-cell activation and anticancer immunity in preclinical studies. For example, the work by Zhou et al. demonstrated that PD-1 aptamers, through their specificity, inhibited the PD-1/PD-L1 interaction, leading to cytokine production and T-cell proliferation in murine cancer models [17]. In another study, a PD-L1 aptamer coupled with nanoparticles for targeted delivery was developed, and preclinical models showed enhanced tumor suppression [67]. In animal models, CTLA-4 aptamers have shown promise in boosting immune responses and slowing tumor growth when explored as alternatives to anti-CTLA-4 antibodies. According to a study by Lee et al, a CTLA-4 aptamer was shown to suppress regulatory T-cell activity and enhance anticancer immunity in melanoma models [68]. To further improve treatment effectiveness, some research has focused on developing bispecific aptamers that target multiple immune checkpoints, such as PD-1 and CTLA-4. These bispecific aptamers have demonstrated synergistic effects in preclinical cancer models [69,70]. In preclinical studies, TIM-3 aptamers have been shown to enhance immune responses and reverse T-cell exhaustion in cancer models, particularly when used alongside other checkpoint inhibitors, such as PD-1/PD-L1 blockers. For instance, TIM-3-targeting aptamers improved anti-tumor immunity in murine models [26]. To further enhance T-cell activation and anti-tumor immunity, LAG-3 aptamers have been developed to block its interaction with MHC class II molecules. Preclinical studies have demonstrated that LAG-3 aptamers work in synergy with PD-1 inhibition to enhance therapeutic outcomes in cancer models [27].

The field of immune checkpoint aptamers is advancing rapidly, with promising preclinical results and early-stage clinical trials. Even though ongoing challenges, their unique characteristics make them a viable alternative to traditional antibody-based therapies. Realizing their full potential in cancer immunotherapy will require further research and clinical validation.

### 3.5. Emerging Targets in Tumor Immunotherapy

In addition to established checkpoints, the exploration of novel immune checkpoints has emerged as a focus in immunotherapy research. Targets such as TIGIT, VISTA, and B7-H3 are being investigated for their roles in immune regulation and tumor progression. Aptamer development for these emerging targets remains in its early stages but shows significant promise. For example, TIGIT is an inhibitory receptor present on both T and NK cells. It interacts with CD155 and CD112 on antigen-presenting cells (APCs) and cancer cells. Regulatory T lymphocytes (TREGs) are stabilized by TIGIT, which inhibits the PI3K/AKT/mTOR pathway, then inhibiting T-cell activation and facilitating immune evasion [71]. TIGIT blockade in combination with radiotherapy has been investigated in both cancer tumor models and in mouse models through activation of the cGAS-STING pathway, induction of chemokine expression, and release of nuclear high-mobility protein from the box-1 group. The study by Zhao and collaborators highlighted the increase in TIGIT/CD155 expression in T cells and DCs after irradiation [62]. Aptamers targeting TIGIT can block its interaction with CD155/CD112, thereby preventing TIGIT-mediated inhibition of T cells and NK cells. This boosts anti-tumor immune responses and makes TIGIT a top candidate for aptamer-based blockade. Early studies suggest that TIGIT aptamers could offer a cost-effective and less immunogenic alternative to monoclonal antibodies (mAbs).

VISTA, an immune checkpoint protein found on both myeloid cells and T cells, functions as an inhibitory regulator of T-cell activation, promoting immune tolerance. Aptamers targeting VISTA can block its inhibitory signals, restoring T-cell activation and enhancing anti-tumor immunity. Similarly, B7-H3 is a co-inhibitory molecule found on both tumor cells and immune cells. It inhibits T-cell activation and contributes to tumor immune evasion. Aptamers targeting B7-H3 can block its interaction with T-cell receptors, reversing immune suppression and enhancing anti-tumor responses [4,72]. Aptamers targeting VISTA and B7-H3 are being designed to disrupt immune suppression and improve anti-tumor immunity. Bi et al. highlight the scalability of aptamer synthesis as a critical advantage in developing therapeutics for emerging targets. Furthermore, the ability to conjugate aptamers with imaging agents or therapeutic payloads provides a unique opportunity for personalized medicine, enabling both targeted therapy and real-time monitoring of treatment efficacy [9].

In general, most immunotherapies targeting these checkpoints are still in preclinical or early-phase clinical trials. While the therapeutic potential of aptamers targeting TIGIT, VISTA, and B7-H3 is being explored in cancer immunotherapy, the data available on these aptamers is limited. Further investigation and clinical development are required to realize their potential fully.

## 4. Multifunctional Aptamers in Tumor Immunotherapy

The development of multifunctional aptamers represents a significant advancement in tumor immunotherapy, offering versatility, specificity, and improved therapeutic outcomes. Multifunctional aptamers utilize their structural flexibility and chemical modifiability to address the complex nature of tumor immunology, including immune evasion, drug resistance, and poor tumor targeting. This section explores the potential of bispecific and multivalent aptamers, aptamer–drug conjugates (ApDCs), and aptamer–nanoparticle conjugates in advancing cancer treatment.

### 4.1. Bispecific and Multivalent Aptamers

Bispecific and multivalent aptamers are designed to simultaneously target two or more molecules, thereby enhancing therapeutic efficacy. These aptamers are engineered to address the redundancy and complexity of signaling pathways in tumor immunology. By inhibiting multiple immune checkpoints at once, bispecific aptamers can increase T-cell activation and sustain anti-tumor immunity. A compelling example of a bispecific aptamer targets both PD-1 and CTLA-4, as highlighted by Bertrand et al. This dual-target approach improves immune activation by concurrently blocking key immune-suppressive pathways. In a separate study, Thomas et al. demonstrated that a bispecific aptamer targeting PD-1 and CTLA-4 provided better tumor suppression than monovalent aptamers or antibody-based treatments in animal models [73]. Similarly, bispecific aptamers targeting PD-1 and TIM-3 have shown improved efficacy in reversing T-cell exhaustion in preclinical studies [10]. Another study found that bispecific aptamer probes significantly enhanced the selective and efficient regulation of receptor activation and downstream signaling cascades. Compared to single-aptamer-mediated regulation, this study presented a diverse framework for designing molecular mediators capable of modifying receptor function, paving the way for innovative therapeutic development [74]. Furthermore, aptamers have been developed for binding to neurologic disease-relevant proteins and suppressing neurodegenerative processes caused by post-translational modifications (such as phosphorylation) and preventing aggregation. These aptamers have shown promise as precise therapies and early diagnostic biomarkers for neurological and neurodegenerative diseases, such as Alzheimer’s disease, prion protein diseases, Huntington’s disease, transmissible spongiform encephalopathies, and Parkinson’s disease [75].

On the other hand, multivalent aptamers enhance binding avidity and increase target engagement. These aptamers are particularly effective within the tumor microenvironment, where low ligand concentrations often limit the efficacy of monovalent therapeutics. For example, a multivalent PD-L1 aptamer developed by Ayass et al. demonstrated improved tumor penetration and enhanced therapeutic activity compared to its monovalent counterpart [5]. Sun and his team developed multivalent aptamers targeting the spike protein of SARS-CoV-2 to prevent the virus from entering host cells. The multivalent design showed promise for antiviral treatment by increasing binding affinity and neutralizing effectiveness [76]. In another separate research, multivalent aptamers that target IL-6 and TNF-α were designed to control inflammatory reactions in autoimmune disorders. The multivalent architecture enhanced the anti-inflammatory benefits in rheumatoid arthritis by enabling the simultaneous suppression of numerous cytokines [69]. In another study, multivalent aptamers that target IL-6 and TNF-α were intended to control inflammatory responses in autoimmune disorders. The multivalent architecture enhanced the anti-inflammatory benefits in rheumatoid arthritis by enabling the simultaneous suppression of multiple cytokines [69]. In research by Chen et al., multivalent aptamers demonstrated increased binding avidity and anticoagulant activity, showing potential for treating cardiovascular diseases [77]. Further studies on multivalent aptamers, as highlighted by McNamara et al., revealed the discovery of an aptamer that binds to 4-1BB, a protein produced on the surface of activated mouse T cells. Multivalent aptamer configurations were shown to enhance T-cell activation in culture and induce tumor rejection in rats [78].

Both multivalent and bispecific aptamers, such as those targeting both CTLA-4 and PD-1, demonstrate enhanced therapeutic efficacy by simultaneously blocking multiple pathways. Studies have shown that these approaches improve T-cell activation compared to monoclonal antibodies. Similarly, ApDCs have shown potential in preliminary cancer models by combining precise targeting with localized drug delivery, thereby reducing off-target effects. While issues in scalability, stability, and regulatory approval remain, future studies should emphasize converting these modified aptamers into therapeutically feasible treatments.

### 4.2. Aptamer–Drug Conjugates (ApDCs)

ApDCs combine the specificity of aptamers with the cytotoxic efficacy of chemotherapeutic agents. This combination enables targeted drug delivery, minimizing off-target effects and improving therapeutic indices. ApDCs are designed to selectively bind tumor-specific markers, ensuring that the attached chemotherapeutic agents are delivered directly to cancer cells. A notable example is the conjugation of a PD-L1 aptamer with doxorubicin, a widely used chemotherapeutic agent. Studies by Bi et al. demonstrated that this ApDC significantly reduced tumor growth in murine models while sparing normal tissues. Another innovative application involves conjugating aptamers with immune-modulating agents, such as interleukin-2 (IL-2), to enhance the immune response against tumors. Case studies in tumor immunotherapy also emphasize the potential of ApDCs in overcoming drug resistance. For instance, an ApDC targeting TIM-3 conjugated with paclitaxel showed promising results in reversing resistance in ovarian cancer models [6]. In a study by Huang et al., the anticancer drug doxorubicin (Dox) was covalently coupled to the DNA aptamer sgc8c. The sgc8c–Dox conjugate selectively targeted and destroyed CCRF-CEM cells (a T-cell acute lymphoblastic leukemia model). The findings revealed that the sgc8c–Dox conjugate exhibited characteristics similar to the sgc8c aptamer, including a significant binding affinity (K(d) = 2.0 ± 0.2 nM) and the ability to be efficiently internalized by target cells [79]. In a separate study, the AS1411 aptamer was linked to doxorubicin using an acid-labile linker, allowing the drug to be released in the acidic environment of cancer cells. In vitro experiments showed preferential uptake and cytotoxicity in nucleolin-positive cancer cells (e.g., MCF-7 cells), while in vivo studies in xenograft mouse models demonstrated a significant reduction in tumor growth compared to free doxorubicin, with lower systemic toxicity [80]. In the field of gene therapy, adeno-associated virus 2 (AAV2) vectors were combined with several sgc8 aptamers, resulting in a significantly improved sgc8–AAV2 construct. The transport efficiency of AAV2 was evaluated using a GFP gene model. Compared to single-aptamer–AAV2 constructs, the G–sgc8–AAV2 vectors demonstrated a 21-fold increase in binding capacity and an enhanced ability to protect sgc8 aptamers from nuclease degradation in PTK7-expressing cells [81]. For radiotherapy, aptamers show promise for the targeted delivery of medicinal radioactive substances. An example of this is a study where an anti-PSMA aptamer was linked to PEGylated liposomes and filled with α-particle-generating actinium-225 (Ac) for targeted delivery to PSMA-positive cancer cells. In vitro studies showed that these conjugates specifically delivered 225Ac and destroyed prostate cancer cells expressing PSMA [82]. In phototherapy, the targeted delivery of phototherapeutic agents can reduce toxicity to healthy tissues by precisely controlling external light sources. For example, in the work carried out by Kruspe et al, an ApDC of the photosensitizer chlorin e6 (Ce6) was created using an aptamer for the interleukin-6 receptor. The resulting ApDC was selectively delivered to and absorbed by the targeted cells, and light exposure induced cell death [83].

These findings highlight the versatility and scientific potential of ApDCs in addressing unmet needs in cancer therapy. However, improving stability, pharmacokinetics, and large-scale manufacturing presents difficulties. With ongoing invention, ApDCs might become a major actor in precision medicine, providing safer and more efficient therapy options for various diseases.

### 4.3. Aptamer–Nanoparticle Conjugates

Aptamer–nanoparticle conjugates leverage the high surface-area-to-volume ratio and tunable properties of nanoparticles to enhance drug delivery and immunotherapy outcomes. Nanoparticles serve as carriers for aptamers, drugs, and imaging agents, facilitating targeted delivery and controlled release within the tumor microenvironment. Gold nanoparticles (AuNPs) conjugated with PD-1 aptamers represent a promising approach to enhancing immune checkpoint blockade. Melian et al. demonstrated that these conjugates not only improved tumor targeting but also enabled real-time imaging of therapeutic efficacy [10]. Similarly, liposomal nanoparticles functionalized with TIM-3 aptamers have been shown to enhance the delivery of siRNA and chemotherapeutics to tumor sites. Another innovative application involves using magnetic nanoparticles conjugated with LAG-3 aptamers for hyperthermia-based cancer treatment. These conjugates enable specific targeting and thermal ablation of tumors, as reported by Ayass et al. The pre-conjugation process involves mixing aptamer–lipid combinations with additional lipids during the liposome formation process, resulting in a one-step manufacturing method. For instance, Hong et al. developed a bispecific α-Gal liposome with aptamer integration using a hydration lipid film and Apt–cholesterol conjugates. This approach activates the immune system to attack tumor cells, leading to their lysis through cell-mediated cytotoxicity dependent on antibodies [84]. To enhance targeted immunity against tumors by CD8+ T lymphocytes, Lu’s group created immuno-activated T cells modified with aptamer-decorated liposomes. By increasing FC/PD-1-CTL penetration in tumor tissue and boosting the systemic immunoactivation levels of cytokines, hEnd-Apt/CD3-Lipo-modified FC/PD-1-CTLs showed improved tumor-fighting capacity and survival rates in vivo in tumor xenograft animal studies [85].

Additionally, a study by Bagalkot et al. reported a targeted cancer imaging, treatment, and sensing tool based on an innovative quantum dot (QD)–aptamer (Apt)–doxorubicin (Dox) conjugate [QD-Apt(Dox)]. Functionalizing the surface of fluorescent QDs with the A10 RNA aptamer, which targets the extracellular region of prostate-specific membrane antigens (PSMA), demonstrated effective targeting and therapeutic potential [86].

Aptamer–nanoparticle conjugates also offer the possibility of combination treatments, where multiple agents are delivered simultaneously to synergize therapeutic outcomes. The schemes below highlight the versatility of multifunctional aptamers as powerful tools for advancing targeted cancer therapy and theranostics. Future studies should focus on improved biostability, pharmacokinetics, toxicity, and long-term effects of aptamers. As summarized in Table 3, recent research has demonstrated the effectiveness of aptamers that target immune checkpoints, such as PD-1, CTLA-4, and TIM-3. Aptamers show promise in enhancing immune responses in the context of cancer immunotherapy.

## 5. Challenges and Limitations in Aptamer Development

Aptamers hold tremendous promise in tumor immunotherapy due to their specificity, versatility, and low immunogenicity. These aptamers include those that target immune checkpoint molecules such as PD-1/PD-L1, TIM-3, LAG-3, and CTLA-4. However, several challenges and limitations remain in their development and application. By addressing these limitations through advancements in aptamer engineering and exploring future directions, aptamers have the potential to revolutionize cancer treatment strategies. Here, we outline these limitations and difficulties. Immune checkpoint molecules are membrane-bound proteins with complex tertiary structures. Their glycosylation sites and conformational flexibility can make it more difficult for aptamers to bind effectively. Furthermore, aptamers need to attach to specific epitopes on these proteins to prevent their interactions successfully. However, it may be challenging to develop high-affinity aptamers due to the structural complexity of these targets [100]. In Anderson et al., the redundancy in immune checkpoint pathways was highlighted, showing that the expression of immune checkpoint molecules in tumors is often heterogeneous, resulting in varying responses to aptamer-based treatments. As cancer cells can upregulate alternative checkpoints, such as TIM-3 or LAG-3, aptamers targeting a single checkpoint (e.g., PD-L1/PD-1) may not be sufficient to overcome immune evasion mechanisms [101]. Like other aptamers, immune checkpoint aptamers are limited by their susceptibility to nuclease degradation in vivo, which shortens their half-life and reduces therapeutic efficacy. Nuclease degradation is common in nucleic acid-based aptamers when exposed to biological fluids. Strategies such as chemical modifications, including the incorporation of 2′-fluoro or 2′-O-methyl groups, have been employed to enhance aptamer stability [5]. Keefe et al. discussed the challenges of optimizing aptamer stability and pharmacokinetics. Chemical modifications, such as 2′-O-methyl or 2′-fluoro replacements, are often necessary to improve stability. However, these modifications can affect binding affinity and specificity [16]. The disadvantages of single-nucleic acid aptamers include poor cellular internalization and quick degradation by nucleases. To solve this disadvantage, the research group of Liu et al. reported that nanomaterials owning fluorescence capabilities, such as QDs, MXene, and UCNPs, were incorporated into the formulation of aptamer nano sensors [102]. However, the degradation processes and long-term safety of nanomaterials, such as PDA, GO, TMDCs, MOF, AuNPs, and QD, remain unidentified. Prodeus et al. emphasized the importance of minimizing off-target effects in aptamer design, as aptamers have the potential to bind unwanted targets, leading to toxicity and off-target consequences. Additionally, immune checkpoint molecules are expressed on both tumor cells and normal immune cells, raising concerns about autoimmune-like side effects [38]. Sun et al. addressed the difficulties of aptamer delivery and the need for innovative strategies to improve tumor penetration. A significant challenge is efficiently delivering aptamers to tumor sites, particularly in solid tumors with poor vascularization [45]. Moreover, tumor resistance to aptamers presents a real challenge. For example, Sharma et al. described the pathways of immune checkpoint blockade resistance and the need for combination therapies. They found that tumors can develop resistance to aptamer-based therapies by downregulating the target checkpoint molecule or upregulating compensatory pathways. This resistance can limit the long-term efficacy of aptamers targeting PD-1/PD-L1, CTLA-4, LAG-3, or TIM-3 [103]. Furthermore, aptamers’ binding affinity and stability need to be strengthened. The majority of aptasensors utilized monovalent aptamers as capture probes. Temperature, pH, ionic strength, interference, viscosity, and other sample variables may readily influence the selectivity and affinity of monovalent aptamers. To address this issue, several strategies were mentioned. Liu et al. reported strategies including the use of multivalent spatial recognition patterns, modification of aptamers, and further optimization of the SELEX procedure [104]. In the construction of aptamer SELEX and aptasensors, microfluidics improves the efficiency and reduces the complexity of the SELEX process, promotes the immediacy and stability of the biosensor response, and integrates with mobile phone software for signal analysis and acquisition. However, microfluidics is still in the early stages of development and is immature and imperfect; for instance, microfluidic aptasensors technology has a wide scope of development in the field of synthetic biology. Developments in computational programs, genome editing, and plasmid technology have expanded the ability to generate a large number of different mutants, but high-throughput biosensor assays for the detection of target-secreting cells are still limited. Aptamers that target small molecules have wide application prospects for the development of new drugs, treating tumors, diagnosing diseases, monitoring environmental pollution, detecting drugs, and sensitive detection applications. However, the simple structures and low molecular masses of small molecules, along with the limited number of binding groups available for interacting with nucleic acids, lead to unstable aptamer–small molecule binding, which presents significant challenges for aptamer screening and sensor development. To address this challenge, Hu et al. reported that efficient screening techniques are critical for identifying aptamers with excellent performance characteristics, [105].

Another significant challenge lies in regulatory and manufacturing hurdles. Unlike monoclonal antibodies, which have well-established pipelines from discovery to commercialization, aptamer manufacturing must overcome less mature production protocols. Consistency in large-scale synthesis, stringent quality control, and regulatory approval processes present obstacles to market access [9]. Moreover, the cost-effectiveness of aptamers, often cited as a benefit, diminishes if manufacturing inefficiencies persist. Nimjee et al. highlighted the challenge of scaling up aptamer production for clinical use. As mentioned in the study, it can be technically challenging and costly to produce aptamers with high purity and consistency on a large scale, and regulatory requirements for manufacturing procedures may cause delays in clinical translation [61]. In a separate study, Sundaram et al. discussed the regulatory challenges associated with aptamer-based therapeutics. They reported that the development process for aptamers may be prolonged due to stringent regulatory criteria for safety and efficacy. Since aptamers targeting immune checkpoints (e.g., PD-1/PD-L1, CTLA-4, LAG-3, and TIM-3) have limited clinical data, predicting their performance in human trials remains challenging [106].

In spite of these challenges, aptamers targeting immune checkpoint molecules hold significant promise for cancer immunotherapy. Successful research and clinical translation will depend on addressing these limitations with novel approaches, such as combination therapies, improved delivery systems, and advanced chemical modifications. Several modifications have been investigated in order to improve bioavailability and increase the stability of aptamers against nuclease degradation, as outlined in Table 4. These include 2′-fluoro (2′-F), 2′-O-methyl (2′-OMe), and terminal PEGylation to prolong circulation time.

## 6. Future Directions and Advancements in Aptamer Engineering

Recent advancements in aptamer engineering are paving the way for overcoming existing challenges. Enhanced selection methods, such as cell-systematic evolution of ligands by exponential enrichment (cell-SELEX), enable the generation of aptamers with high specificity and affinity for tumor-related targets. For instance, Melian et al. proposed the development of aptamers with improved binding properties by using modified SELEX protocols tailored for immune checkpoint molecules. Although the SELEX technique has greatly improved, identifying aptamers experimentally remains challenging and time-consuming. Structure-based approaches are increasingly popular in computer-aided aptamer design and development. For instance, Fallah et al. employed a structure-based method to develop aptamers with high affinity [115]. Similarly, to address the time-consuming and challenging nature of the SELEX method, Sun et al. used computational tools for aptamer detection and refinement [116].

Artificial intelligence (AI) and computational biology have emerged as game-changers in aptamer design. To predict the binding affinity of aptamers for their targets, AI techniques, such as machine learning (ML) and deep learning (DL), are employed. Large datasets of aptamer–target interactions are used to train these models, which identify patterns and optimize sequences. For example, a deep learning algorithm was employed by Wu et al. to predict aptamer sequences targeting cancer biomarkers. The algorithm successfully identified high-affinity aptamers targeting tumor-specific proteins, having been trained on a dataset of aptamer–protein interactions [117]. In a separate study, Emami et al. introduced AptaNet, a deep neural network that predicts aptamer–protein interaction pairs by combining data obtained from target proteins and aptamers. The study used two encoding methods: k-mer and inverse complementary k-mer frequencies to encode the aptamers. The findings suggest that AptaNet can help identify new aptamer–protein pairs and improve understanding of the relationship between aptamers and proteins [118]. Similarly, a study by Yang et al. employed machine learning to predict aptamer–protein interactions by combining sequence characteristics from aptamers and target proteins. Aptamers were identified based on their amino acid compositions and pseudo-K-tuple nucleotide structures. Additionally, sparse autoencoders were used to characterize the target protein sequence. The results showed that this model could identify novel aptamer–protein interaction pairs and uncover the relationship between aptamers and proteins [119]. To identify highly accurate and efficient nucleic acid ligands, Song et al. developed SMART-Aptamer, a sequential multidimensional analysis approach for aptamer identification based on unsupervised machine learning and multilevel structural analysis. Using high-throughput sequencing (HTS) data from SELEX libraries, SMART-Aptamer successfully identified aptamers with high affinity for three target sets (hESCs, EpCAM, and CSV), with minimal false positives and negatives [120].

Machine learning algorithms can predict aptamer–target interactions, optimize secondary structures, and identify novel target molecules, greatly accelerating the discovery process. These tools allow researchers to screen large libraries efficiently, reducing the reliance on labor-intensive experimental techniques. Computational methods also facilitate the design of multifunctional aptamers, such as bispecific and multivalent constructs, by modeling complex interactions between aptamers and their targets [6]. The integration of aptamers into personalized medicine represents a transformative possibility in tumor immunotherapy. Personalized medicine focuses on tailoring treatments to individual patient profiles, which aligns well with the customizable nature of aptamers. For example, patient-specific aptamers can be designed to target particular tumor antigens or immune checkpoint profiles, enhancing therapeutic efficacy and minimizing off-target effects.

Combination therapy strategies involving aptamers offer another promising approach. Aptamers can be used alongside other immunotherapeutic agents, such as monoclonal antibodies or immune checkpoint inhibitors, to synergize anti-tumor responses. Ayass et al. highlighted the potential of combining PD-1 aptamers with chemotherapeutic agents in aptamer–drug conjugates (ApDCs) to achieve improved outcomes in preclinical models [5]. Similarly, aptamer–nanoparticle conjugates could serve as carriers for delivering a combination of drugs, immunomodulators, or genetic material directly to the tumor microenvironment.

Aptamer-based biosensors for early cancer detection utilize technologies such as electrochemical sensors, enabling real-time biomarker detection. Integrating aptamers into personalized medicine through combinatory approaches with immune checkpoint inhibitors or chemotherapies holds great promise, as highlighted by recent studies demonstrating enhanced efficacy in resistant cancers. Some specific research and technologies related to aptamer-based biosensors for cancer detection. For example, electrochemical biosensors are frequently used to detect cancer biomarkers. When aptamers are immobilized on electrode surfaces and bind to cancer biomarkers, electrochemical changes are detected. For instance, to detect MUC1, a protein biomarker overexpressed in several malignancies, Quazi et al. designed an electrochemical aptamer-based biosensor that demonstrated good selectivity and a detection limit of 0.0038 pM in breast cancer specimens [121]. In contrast, aptamers tagged with quantum dots or fluorescent dyes are used in fluorescence biosensors. The fluorescence intensity changes as the aptamers bind to cancer biomarkers. A compelling example of a fluorescent aptamer-based biosensor was designed by Zhang et al. to detect MUC1, which is highly expressed in various cancer cells, especially lung adenocarcinoma. Aptamer-functionalized Zn^(2+)^-dyed CdTe quantum dots (aptamer–QDs) were effectively utilized for active tumor-targeting imaging both in vitro and in vivo. A universal DNA design for the manufacture of Zn^(2+)^-dyed CdTe QDs can be applied to various target sequences. Due to their specific identification capabilities and straightforward synthesis pathway, the produced QDs are compact in size (3.85 ± 0.53 nm), with excellent quantum yield (greater than 80.5%) and outstanding photostability. Furthermore, the toxic impact of QDs was significantly reduced due to Zn-dying and the presence of DNA [122]. In a preclinical model (HeLa cells), AS1411 aptamers targeting nucleolin were labeled with magnetic nanoparticles (NPs) to evaluate the toxicity, biological distribution, and targeted T1 magnetic resonance imaging (MRI) in human cervical cancer tumor-bearing mice. The resulting NPs were referred to as Mn_3_O_4_@SiO_2_(RB)-PEG-Apt. In vivo MRI and in vitro fluorescent confocal imaging showed that the nanoparticles successfully targeted cancer cells and exhibited significant tumor accumulation. A quantitative biodistribution analysis corroborated the imaging results [123]. Colorimetric biosensor technology is suitable for point-of-care applications because it uses aptamers to induce detectable color changes when cancer biomarkers are present. It is also suitable for food safety testing. Geleta et al. developed an exemplary colorimetric aptamer-based biosensor to detect toxic substances generated by microbes, including algae, fungi, and bacteria. Colorimetric detection methods have proven superior to conventional techniques due to their user-friendliness, rapid response, cost-effectiveness, and visibility to the human eye [124]. While detecting cancer biomarkers in small sample volumes with high throughput can be complex, Sheng et al. engineered a microfluidic system using aptamers to sort and analyze various cell types. The system successfully isolated target leukemia cells from cellular mixtures, achieving around 95% capturing accuracy and 81% purity, capturing as few as 10 tumor cells from 1 mL of total blood volume. The study also addressed the issue of low throughput in conventional microfluidic devices by processing 1 mL of blood in just 28 min [125]. Surface-patterned aptamers are used to capture specific cells in microchambers with a temperature regulation apparatus. Specific cell release is achieved using a set of microheaters and temperature sensors to minimize temperature fluctuations, thus breaking the cell–aptamer connections within a designated zone. Experiments with CCRF-CEM cells and sgc8c aptamers demonstrated targeted cell entrapment and temperature-induced release of certain cell populations, resulting in minimal impairment to their survival [126].

Advances in nanotechnology and bioinformatics are expected to play a crucial role in future aptamer applications. For instance, Achiko et al. recently created an integrated platform that combines a DNA aptamer-based diagnostic test with its digital health passporting software for low-cost, quick, saliva-based, and user-friendly detection of SARS-CoV-2 [104]. The development of aptamer-based biosensors for early cancer diagnosis, along with their integration into theranostic systems, could redefine how cancer is diagnosed and treated. These innovations align with the broader vision of precision oncology, where aptamers are fundamental to achieving highly targeted, minimally invasive, and effective cancer therapies.

Finally, to ensure reproducibility, reliability, and safety when using aptamers in clinical settings, standardized protocols must be developed and implemented. Studies should be conducted using rigorous techniques, such as clearly defined patient groups, carefully monitored experimental conditions, and reliable validation procedures. Important outcomes to measure include safety profiles (toxicity and adverse events), therapeutic efficacy (e.g., reduction in disease biomarkers or clinical improvement), pharmacokinetics (absorption, distribution, metabolism, and elimination), and diagnostic accuracy (sensitivity, specificity, and predictive values). To ensure the feasibility of aptamer-based solutions in clinical settings, research should also assess their scalability and cost-effectiveness [127].

## 7. Conclusions

This review provides a comprehensive analysis of the advancements and challenges in employing aptamers for tumor immunotherapy, particularly in the context of immune checkpoint blockade. Through a detailed evaluation of the PD-1/PD-L1 pathway and emerging targets, such as TIM-3, LAG-3, and CTLA-4, we highlight the superior specificity, modularity, and lower immunogenicity of aptamers compared to monoclonal antibodies. Their amenability to chemical modification enables the design of multifunctional formats, including bispecific aptamers, ApDCs, and aptamer–nanoparticle hybrids, significantly expanding their therapeutic landscape.

Despite these advantages, several technical barriers persist, such as in vivo stability and large-scale manufacturing. Our review highlights the importance of advanced SELEX techniques, structure optimization, and AI-driven aptamer discovery in addressing these limitations. Additionally, integrating aptamers into combination immunotherapies presents promising opportunities for personalized oncology.

In summary, aptamers are emerging as powerful tools in cancer immunotherapy, offering targeted and tunable solutions for immune modulation. Continued interdisciplinary research, bridging molecular biology, computational science, and clinical medicine, is essential to realize their full clinical potential. This review serves as a roadmap for future development, directing efforts toward robust design strategies, translational validation, and clinical integration of aptamer-based therapeutics.

## Figures and Tables

**Table 1:** Aptamers and binding targets in cancer immunotherapy.

Targets	Target Cells	Aptamers	Binding Affinities	Functions	Aptamer Sequences	References
PD-L1	Tumor cell	AptPD-L1	~5–50 nM	PD-L1/PD-1 interaction inhibitors	5′-ATACCAGCTTATTCAATTGTAGAGTATAAAAAGAGTGATGATCTTTTGTAGGTTTTTTAGATAGTAAGTGCAATCT-3′	[21]
PD-1	T-cells	PD-1 Apt1	~10–100 nM	PD-L1/PD-1 interaction inhibitors	5′-TCCCTACGGCGCTAACCCTCCCCTAGTATATATTGTCCTCGTCTATGCCACCGTGCTA CAAC-3′	[22]
CTLA-4	T-cells	CTLA-4 Apt	~10–100 nM	Regulate T-cell activation by outcompeting CD28 for B7 binding	5′-GGGAGAGAGGAAGAGGGATAGGCACCGGAAGGGCTACACTCCTATATCCCCTGCCcAGCCCGCCATAACCCAGAGGTCGATAGTACYGGATCCCCCC-3′	[23]
CTLA-4/NKG2A dual receptors	T-cells/NK cells	AYA22T-R2-13	~1–50 nM	Enhancing CD8+ T-cells and NK cells effector functions	5′-ACACdUdUdUdUCCCCCACCdUGAdUCCdUCAGdUdUCCGGAAAAGdUGdU-3′	[5]
Nucleolin	Tumor cells	AS1411	~1–10 nM	Inhibiting cancer cell proliferation	5′- CCAGCCATCCAAAACTCTGTGGTGGTGGTGGTTGTGGTGGTGGTGGTAACTATCCTTGCCCGAACG-3′	[24]
PTK7	Tumor cells	Sgc8	~0.2–2 nM	Inhibit EGFR signaling	5′-ATACCAGCTTATTCAATTAAAGNTAATCGCCGTAGAAAAGCATGTCAAAGCCGGAACCNCAGATAGTAAGTGCAATCT-3′	[25]
TIM-3	T cells	S3.1	~10–20 nM	Block TIM-3/galectin- 9 interaction	5′-GGGGGAATTCTAATACGACTCACTATAGGGAGGACGATGCGGGGGAUGCUCAUUCAACGUUCCAGAUAUCAGGGCAUCCCCAGACGACTCGCTGAGGATCC-3′	[26]
LAG-3	T cells	SL15	~20–200 nM	Block LAG-3/MHC-II interaction	5′-GGGGAATTCTAATACGACTCACTATAGGGAGAGAGATATAAGGGAGAGAATTTGGTAATGGGCCCTTATATCTCTCTCCCATTACCAAATTCTCTCCC-3′	[27]
SDF-1	Tumor cells	NOX-A12 (Spiegelmer)	0.2–0.5 nM	Binds and neutralizes SDF-1 thereby blocking its interaction with CXCR4 and CXCR7	5′-GGCGACAUUGGUGGCUUUCUACUGCUUGUGAGUAUUUCGUACAGCUGCUAUAGUGAGUA-3′	[28]
CXCL12	Tumor cells	CXCL12 Apt (Spiegelmer)	~0.4–1.5 nM	Inhibits CXCL12-mediated chemotaxis and inhibits tumor metastasis	5′-ATGAACGCCAAGGTCGTGGTCTGGCTGTTGTGCTTACTTGTTT-3′	[29]
VEGF	Vascular endothelial cells	Pegaptanib (Macugen^®^)	~0.1–2 nM	Block VEGF interaction with VEGFR2, reducing endothelial cell proliferation	5′-TCGGGCGAGTCGTCTGTAATACGACTCACTATAGGGAGGACGATGCGG(N30or40)CAGACGACTCGCCCGATAATACGACTCACTATAGGGAGGACGATGCGG-3′	[19]
TLR9	B cells	CpG7909	~1–100 nM	Active innate immunity and promotes Th1 responses	5′-CCAGTCGTACAGGAAACATGCGTTCTAGATGTTCGGGGC-3′	[30]
TNF-α	Macrophages	VR11	~1–10 nM	Inhibit TNFα signaling	5′-TGGTGGATGGCGCAGTCGGCGACAA-3′	[31]
IL-6	Macrophages	SL1025	~0.4–9.6 nM	Block IL-6/IL-6R interaction	5′-GATGTGAGTGTGTGACGAGN40CACAGAGAAGAAACAAGACC-3′	[32]
IFN-γ	Macrophages	ARC225	~0.26–10 nM	Activate macrophages	5′-TGCCCGTGTCCCGAGGAGGTGCCCTATTTTGCTTGATTATCTCTAAGGGATTTGGGCGG-3′	[33]

SDF-1—stromal cell-derived factor-1; PTK7—protein-tyrosine kinase 7; VEGF—vascular endothelial growth factor; TLR9—toll-like receptor 9; TNF-α—tumor necrosis factor-α; IL-6—interleukin-6; IFN-γ—interferon-γ; CXCL12—chemokine (C-X-C motif) ligand 12; LAG-3—lymphocyte-activation gene; MHC-II—major histocompatibility complex class II molecules; TIM-3—T-cell immunoglobulin- and mucin-domain-containing protein 3; NKG2A—natural killer group 2 member A; HLA-E—human leukocyte antigen E; CTLA-4—cytotoxic T lymphocyte-associated antigen 4.

**Table 2:** Comprehensive comparison of PD-1/PD-L1 aptamers and monoclonal antibodies.

Parameter	Monoclonal Antibodies (mAbs)	PD-1/PD-L1 Aptamers
Therapeutic efficacy	Proven efficacy in blocking PD-1/PD-L1 interaction, causes sustained anti-tumor reactions in cancers like melanoma and NSCLC [6]	Comparable blocking of PD-1/PD-L1 interaction, with enhanced tumor penetration [5]
Safety profile	Associated with immune-related adverse events (irAEs), including colitis and pneumonitis, due to broad immune activation [8]	Minimal irAEs and off-target effects; lower risk of systemic immune activation and reduced likelihood of allergic reactions [10]
Immunogenicity	High immunogenicity, as antibodies are protein-based, leading to potential allergic reactions and anti-drug antibody responses [6]	Low immunogenicity due to their non-proteinaceous, synthetic nature, making them safer for repeated use [9]
Production cost	High cost due to complex bioprocessing in mammalian cell cultures, requiring stringent quality control [9]	Significantly lower cost due to entirely chemical synthesis, with easier scalability and lower material costs [5]
Development timeline	Longer timelines are driven by the complexities of antibody discovery, optimization, and cell-based production [8]	Synthetic design and high-throughput screening techniques enable faster timelines [10]
Stability and handling	Requires refrigeration and controlled conditions to maintain bioactivity, with limited stability outside cold-chain logistics [6]	Chemically modifiable for enhanced stability; can withstand harsher conditions and longer storage periods [9]
Cost to patients	Expensive, with treatment costs ranging between $100,000–$150,000 per patient annually, limiting accessibility [5]	Lower cost per treatment cycle, making them more affordable and accessible, especially in low-income settings [10]
Clinical accessibility	Widely approved and available for various cancers, forming the backbone of current immunotherapy protocols [6]	Preclinical and early clinical stages; promising results suggest potential for future approvals [5]
Tissue penetration	Limited penetration in dense tumor microenvironments due to large molecular size [8]	Superior penetration in solid tumors due to smaller size and enhanced molecular flexibility [10]
Environmental impact	High environmental impact due to reliance on biologics manufacturing facilities and extensive resource consumption [9]	Low environmental impact due to pure chemical synthesis processes and reduced reliance on animal-based production systems [5]

**Table 3:** Aptamers in immune checkpoints for immunotherapy.

Focus	Key Findings/Contributions	Relevance	References
Dual checkpoint aptamer immunotherapy targeting CTLA-4 and NKG2A	Demonstrated enhanced efficacy in tailored cancer treatment using aptamer-based dual checkpoint targeting	Advances in tailored cancer immunotherapy strategies	[5]
A highly specific aptamer probe targeting PD-L1 in tumor tissues	High PD-L1 specificity of aptamer probe mutations in the aptamer sequence can increase its specificity	Making aptamer-targeted therapies may aid personalized medicine approaches	[87]
Tumor and immune reprogramming in advanced renal cell carcinoma	Identified molecular and immune changes during immunotherapy	Insights into the mechanisms of immunotherapy responses	[9]
Aptamer therapy in triple-negative breast cancer	Found that aptamer-targeted therapy enhances immune checkpoint blockade	Supports aptamer use in potentiating immunotherapy	[52]
Circular bispecific aptamer-mediated artificial intercellular recognition for targeted T cell	T cell accumulation and activation improved with reduced complexity and time	Create a new T cell “recognition activation” strategy without ex vivo engineering	[88]
Advancing cancer immunotherapy	Summarizes recent progress and future directions in cancer immunotherapy	Visionary insights for the immunotherapy field	[89]
Dogs in cancer immunotherapy research	Proposed dogs as model organisms for translational cancer immunotherapy	Broadens the scope of animal models in cancer research	[90]
Bispecific aptamer targeting PD-1 and nucleolin	Demonstrated anti-tumor efficacy and immune modulation in vitro and in vivo	Highlights bispecific aptamers as innovative cancer therapies	[69]
Bispecific aptamer enhances immune cytotoxicity against MUC1-positive tumors	A novel approach to targeting antitumor immune reactions against MUC1	Develop a bispecific aptamer targeting MUC1 (tumor marker) and CD16 (on immune cells	[91]
Bispecific aptamer-based recognition-then-conjugation strategy for PD1/PDL1 axis blockade and enhanced immunotherapy	The recognition-then-conjugation strategy may boost tumor immunity	Develop a bispecific aptamer-based PD1/PDL1 axis blocker to improve immunotherapy	[92]
Bispecific nanobody-aptamer conjugates for enhanced cancer therapy	Strong steric hindrance, high affinity, and specificity for tumor cells expressing both targets	Target two distinct antigens or receptors on cancer cells simultaneously	[93]
CAR-aptamers enable traceless enrichment and monitoring of CAR-positive cells	Enriching CAR-T cells efficiently and cheaply, monitoring expansion in vivo, and overcoming tumor escape	Design bispecific circular aptamers to retarget CAR-T cells to tumors after antigen loss	[94]
Nanotherapeutics functionalized with PD-1/PD-L1 aptamers	Developed functionalized nanoparticles for cancer immunotherapy.	Advances in nanotechnology in immunotherapy applications	[67]
Immune checkpoint inhibitors via aptamers	Highlights recent progress in aptamer-based checkpoint inhibitors	Explores alternative checkpoint inhibitors	[4]
Targeting the PD-1/PD-L1 immune evasion axis with DNA aptamers as therapeutics	PD-1 antagonistic aptamers may be a better alternative to antibody-based therapies	Produce synthetic DNA aptamers that bind specifically to the murine extracellular domain of PD-1 and inhibit interaction	[38]
Cell-based cancer immunotherapy via stem cell engineering	Developed engineered stem cells for enhanced immunotherapy	Advances in cellular approaches in cancer therapy	[95]
A novel PD-L1-targeting antagonistic DNA aptamer with antitumor effects	Increased lymphocyte proliferation in vitro and inhibited tumor growth	Develop DNA aptamer blocks PD-1/PD-L1 interaction and targets PD-L1.	[96]
Novel complex of PD-L1 aptamer and albumin enhances antitumor efficacy	A promising strategy to improve aptamer in vivo functionality and which may be useful in immunotherapy	Test PD-L1 aptamer albumin complex could improve antitumor efficacy in vivo	[97]
Dual inhibitory aptamer-ASO delivery system	Anti-tumor efficacy of a dual-functional delivery system	Combining aptamers and ASOs in therapy	[98]
Isolation of DNA aptamer targeting PD-1 with an antitumor immunotherapy effect	Highest affinity and restored T cell function suppressed by PD-1/PD-L1	Identify PD-1-targeted DNA aptamers for cancer immunotherapy	[99]

**Table 4:** Modifications over aptamer addressing nuclease degradation and poor bioavailability.

Modification Type	Added Agents	Contributions	References
Chemical modifications	2′-O-Methyl(2′-OMe), 2′-Fluoro(2′-F) and 2′-Amino (2′-NH_2_)	Significantly promote nuclease resistance of aptamer without sacrificing binding affinity.	[16]
	PEGylation	Increase aptamer stability and prolong circulation time in the bloodstream.	[107]
Molecule conjugation	Protein (e.g., albumin)	Enhance aptamer pharmacokinetic properties.	[108]
	Lipid	Enhance aptamer bioavailability and cellular absorption.	[109]
Nanoparticle encapsulation	AuNPs	Improve aptamer stability and cellular uptake.	[110]
	Polymeric and liposomes	Protect aptamer from nuclease degradation and promote delivery to target tissues.	[111]
Spiegelmers	L-enantiomers	Enhanced resistance of aptamer to nuclease degradation.	[112]
Optimization sequence	Computational tools	Aptamer with optimal stability and binding properties.	[113]
Drug conjugation	Therapeutic substances (e.g., Dox and Dau)	Improve aptamer stability and targeted effectiveness.	[114]
